# Impaired Relationship between Sense of Agency and Prediction Error Due to Post-Stroke Sensorimotor Deficits

**DOI:** 10.3390/jcm11123307

**Published:** 2022-06-09

**Authors:** Yu Miyawaki, Takeshi Otani, Shu Morioka

**Affiliations:** 1 Human Augmentation Research Center, National Institute of Advanced Industrial Science and Technology, Kashiwa 277-0882, Japan; 2 Neurorehabilitation Research Center, Kio University, Kitakaturagi-gun 635-0832, Japan; 3 Department of Rehabilitation, Ishikawa Hospital, Himeji 671-0221, Japan; goo.goo.pt4@gmail.com

**Keywords:** sense of agency, prediction error, stroke, motor deficit, self-other attribution, motor control, sensorimotor system

## Abstract

Sense of agency refers to the experience of controlling one’s actions. Studies on healthy people indicated that their self-other attribution can be realized based on prediction error which is an inconsistency between the internal prediction and sensory feedback of the movements. However, studies on patients with post-stroke sensorimotor deficits hypothesized that their self-other attribution can be based on different attribution strategies. This preliminary study examined this hypothesis by investigating whether post-stroke sensorimotor deficits can diminish the correlation between prediction errors and self-other judgments. Participants performed sinusoidal movements with visual feedback and judged if it represented their or another’s movements (i.e., self-other judgment). The results indicated that the patient who had worse upper limb sensorimotor deficits and lesser paretic upper limb activity compared with the other patient made more misattributions and showed a lower correlation between prediction errors and self-other judgments. This finding suggests that post-stroke sensorimotor deficits can impair the relationship between prediction error and self-other attribution, supporting the hypothesis that patients with such deficits can have altered strategies for the registration of agency.

## 1. Introduction

Sense of agency refers to the experience of controlling one’s actions [1]. This sense plays an important role in controlling self-body movements and interacting with the external world, and is therefore widely discussed in terms of its relationship with movement or mental disorders. In terms of motor control, the sense of agency is induced by mainly utilizing sensorimotor cues, including sensory feedback and its internal prediction [2,3]. These cues work through their comparison processes [4,5], wherein sensory feedback is compared with the internal prediction based on an efference copy of the motor commands [6,7]. When it matches the internal prediction, the self-attribution of the sensation is realized, i.e., registration of agency [8,9]. Based on this theory, studies have shown that self-other attributions in healthy people correlated with prediction errors, which are inconsistencies between sensory feedback and its internal prediction [10,11]. Specifically, the larger the prediction error, the more the participants attributed the sensation to the other, whereas the smaller the error, the more they attributed it to the self. These findings suggest that the sense of agency is linked to the sensorimotor system which computes prediction errors [10,12].

Since the sense of agency can be grounded within the sensorimotor system in motor control [3], post-stroke sensorimotor deficits may result in altered self-other attribution (i.e., misattribution) through disturbing the sensorimotor system. Our previous studies examined this possibility [13,14]. The results showed that patients with post-stroke sensorimotor deficits compared with healthy elderly participants, significantly misattributed the cursor movements that were provided to participants as visual feedback of their movements [13]. Furthermore, a longitudinal case study indicated that such misattributions may depend on the sensorimotor deficit severity and paretic upper limb activity [14]. These studies suggested that sensorimotor deficits are involved in making misattributions; however, it remains unclear why such patients made the misattributions and how they made self-other attributions. The researchers hypothesized that patients with post-stroke sensorimotor deficits make self-other attributions based not on sensorimotor cues (i.e., prediction errors), but on other agency cues because of the sensorimotor system being disturbed by their deficits [13,14]. These altered strategies for self-other attribution might result in misattributions.

This hypothesis is based on cue integration theory [2,15]. According to this theory, if available sensorimotor cues are insufficient or noisy, other types of agency cues, such as thoughts or knowledge [16,17,18], can be more weighted and utilized for self-other attribution. In terms of post-stroke sensorimotor deficits, patients receive less or noisy sensorimotor information daily because their deficits disturb sensory input and reduce their paretic upper limb use [19,20]. Because they experience this situation all the time, their attribution strategies might alter such that their self-other attributions are based not on sensorimotor cues but on other agency cues [13]. To investigate this hypothesis, we first need to investigate whether the relationship between sensorimotor cues and self-other attribution is impaired in such patients before investigating other agency cues. A study with healthy people [11] found a relationship between prediction error and self-other attribution by investigating the correlation coefficient between their indexes in a self-other attribution task [10]. According to the study [11], this approach can allow us to test the relationship between sensorimotor cues and self-other attribution in such patients by investigating the correlation between prediction error and self-other attribution indexes that the patients show. This test can contribute to revealing their strategies for self-other attribution.

The present study preliminarily examined the hypothesis, proposed in our previous studies [13,14], by investigating the correlation between prediction error and self-other attribution indexes. These indexes were measured using the self-other attribution task [10] modified for a clinical investigation [13,14]. In this task, participants performed sinusoidal movements and received their visual feedback as cursor movements on a monitor. They were required to judge whether the cursor movement represented theirs or a PC-controlled movement (i.e., self-other judgment) by referring to the online spatiotemporal inconsistency between their and the cursor movement (i.e., prediction error). We expected that the patient with worse upper limb sensorimotor deficits and lesser paretic upper limb activity compared with the other patient would make more misattributions; the patient with milder upper limb sensorimotor deficits and greater paretic upper limb activity, as well as healthy participants, would make fewer misattributions, as shown by our previous studies [13,14]. We also expected that self-other judgments in the former patient would not or be less correlated with prediction errors, whereas those in the latter patient and healthy participants would be more correlated with prediction errors. If this is verified, the finding would suggest that post-stroke sensorimotor deficits can impair the relationship between prediction error and self-other attribution.

## 2. Materials and Methods

### 2.1. Participants

**Figure 1:**
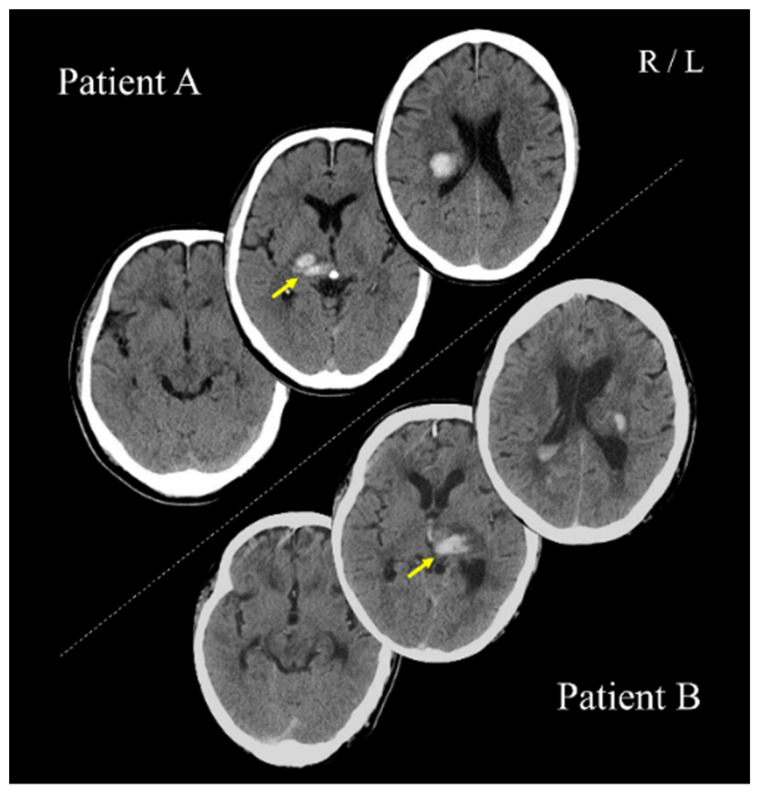
Computed tomography scans show the lesion sites of patients A and B. These images were acquired within a day after stroke onset. Yellow arrows indicate the lesion sites. R: right hemisphere; L: left hemisphere.

Patients A (PA; 70s; male) and B (PB; 60s; male) with post-stroke upper limb sensorimotor deficits, as well as three healthy adults (27–30 years; two women), participated in the study. PA and PB had right and left thalamic hemorrhage strokes, respectively (Figure 1). For PA and PB, 64 and 67 days had passed since stroke onset, respectively. All participants were right-handed. This study was conducted with the approval of the Ethics Committee of the Jinjukai Ishikawa Hospital (2018-1). All participants provided written informed consent.

### 2.2. Assessments of Clinical Characteristics

PA had a paretic left upper limb with moderate sensorimotor deficits and occasionally used it in his daily life, whereas PB had a paretic right upper limb with mild sensorimotor deficits and often used it. Motor deficits were assessed using the Fugl-Meyer Assessment of the upper extremity (FMA-UE) and the Action Research Arm Test (ARAT). The FMA-UE comprises the total score of shoulder/elbow/forearm, wrist, hand, and coordination movement subscales. The ARAT comprises the total score of grasp, grip, pinch, and gross movement subscales. Proprioception deficits were assessed using the shoulder, elbow, wrist, and first finger proprioception subscales of the FMA-UE, and tactile deficits were assessed using the forearm and hand tactile subscales of the FMA-UE. The amount and quality of paretic upper limb use in daily lives were assessed using the Motor Activity Log (MAL). The MAL comprises the amount of use (AOU) and quality of movement (QOM) subscales; the mean score for each subscale was calculated.

PA and PB had no history or diagnoses of any cognitive impairments, psychiatric disorders, or neurological dysfunctions, except for their post-stroke sensorimotor deficits. Cognitive and attention impairments were assessed using the Mini Mental State Examination (MMSE) and the Trail-Making Test A (TMT-A), respectively. Apraxia was assessed using the Korean version of the apraxia screen of TULIA (K-AST; [21]). Anosognosia was assessed through a clinical interview, wherein the clinical expert verified whether they were aware of their sensorimotor deficits and could explain these deficits. Neglect was assessed using the Catherine Bergego Scale (CBS). A summary of patient data is shown in Table 1.

### 2.3. Apparatus

**Figure 2:**
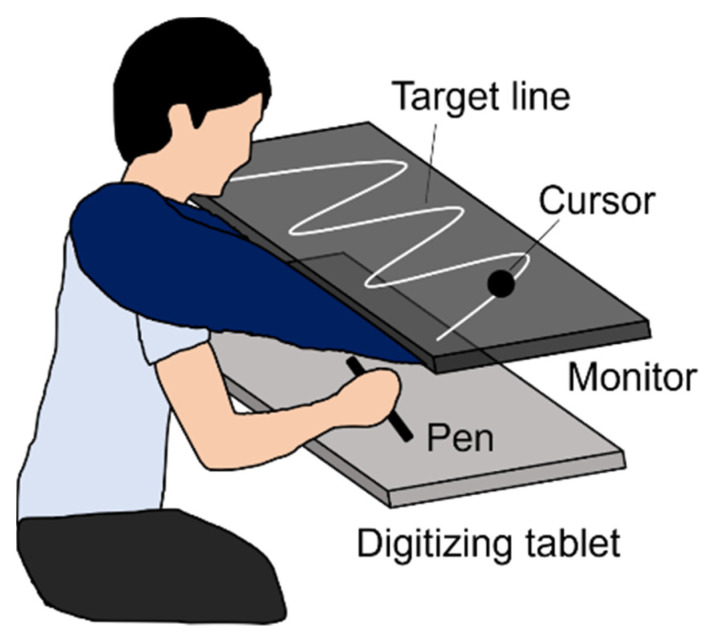
Experimental setup.

A monitor (UQ-PM14FHDNT, UNIQ, Kashiwa, Japan) with a 60 Hz refresh rate was set parallel to and 20 cm above a digitizing tablet (Intuos Pro PTH-860, Wacom, Kazo, Japan), as shown in Figure 2. The plotting area of the monitor (309 × 174 mm) was similar to the input area of the digitizing tablet. A sine wave comprising three cycles was displayed as the target line on the monitor. The experiment was programmed using Hot Soup Processor 3.51 (Onion Software, Japan).

### 2.4. Task

The basic task and procedure followed those of Asai [10], and were simplified to be suitable for the clinical investigation [13,14]. Participants manipulated a pen device on the digitizer to trace the target line as accurately as possible, and received visual feedback as cursor movement (Figure 2). Regarding visual feedback, there were two conditions: SELF and FAKE. In the former, participants received the cursor feedback representing their actual pen movement, and were required to trace the target line by controlling the cursor. In the FAKE condition, they received the cursor feedback representing a prerecorded movement, and were required to trace the target line using their proprioception without visual feedback because they had no control over its cursor movement. The prerecorded movements were participants’ movements that had been secretly recorded in a practice session (see Procedure). Participants were instructed to judge whether the cursor movement represented theirs or a PC-controlled movement (i.e., self-other judgment) by referring to the online spatiotemporal inconsistency between their pen and the cursor movements (i.e., prediction error).

### 2.5. Procedure

PA and PB completed the task using their non-paralyzed upper limb, and healthy participants completed it using their right upper limb. First, participants placed a pen at the start position (i.e., left side of the target line in investigations with PA and healthy participants or right side in the investigation with PB) on the digitizing tablet and maintained the pen position until the countdown reached from three to zero. When zero was displayed, a computer made a short beep sound, and participants started the tracing movement. After that, participants traced the target line toward the goal position (i.e., three cycles of sinusoidal movement) such that the timing of the pen tip reached the peak or trough of the sine wave matched that of a short beep sound provided in one-second intervals. In Cycles 2 and 3, participants were shown the cursor as visual feedback of their movement, and made self-other attribution of the cursor movement. During Cycle 1 and the first 0.5 s of Cycle 2 as well as the last 0.5 s of Cycle 3, the cursor was masked to prevent participants from distinguishing between the self and fake movements by the timing of the start and end of the movements. During these, they were required to trace the target line using their proprioception without visual feedback.

This experiment comprised 32 trials because each of the SELF and FAKE conditions was tested 16 times in random order. Before the main experiment, participants were trained to be familiar with the experimental procedure through three practice sessions. In the first, they became familiar with the required movement and the device by training. In the second, they performed 25 trials of tracing the target line with the cursor representing visual feedback of their movement. The prerecorded movements in the FAKE condition were randomly chosen from the last 20 trials of these practice trials; the first five trials were not used as the prerecorded movements to exclude unstable ones. In the third, they performed four sample trials in each of the SELF and FAKE conditions (i.e., eight trials in total). After these practice sessions, the main experiment started.

### 2.6. Indices

During the movement task, vertical distances between the pen positions and target line were measured as an index of movement error. Although movement error did not function as an index of self-other attribution because the task was simplified [13,14], it was measured to confirm whether participants realized feedback control of the cursor movement. In the FAKE condition, Euclidean distances between the pen and cursor positions (pen–cursor distances) were calculated as an index of prediction error [11]; a previous study showed that pen–cursor distances negatively correlated with self-other judgment scores, indicating that the longer the distances, the more the participants attributed the cursor movement to the other [11]. Hence, we focused on prediction errors and self-other judgments in the FAKE condition. In the SELF condition, participants’ pen positions were consistent with the cursor positions (i.e., no prediction errors in theory). After each trial, participants verbally reported their self-other judgment on a 9-point scale ranging from 9 (completely self-movement) to 1 (completely other’s movement). Their incorrect responses were used as an index of subjective misattribution in self-other attribution [22].

### 2.7. Statistical Analysis

Regarding movement errors and self-other judgments, we visually confirmed participants’ plots. To investigate the correlation between prediction errors and self-other judgments, we confirmed scatter plots with the mean pen–cursor distance and self-other judgment score of each trial in the FAKE condition and conducted correlation analyses to calculate the correlation coefficients between them. Through these correlations, we preliminarily investigated the hypothesis that post-stroke sensorimotor deficits can impair the relationship between prediction error and self-other attribution.

## 3. Results

### 3.1. Movement Error

**Figure 3:**
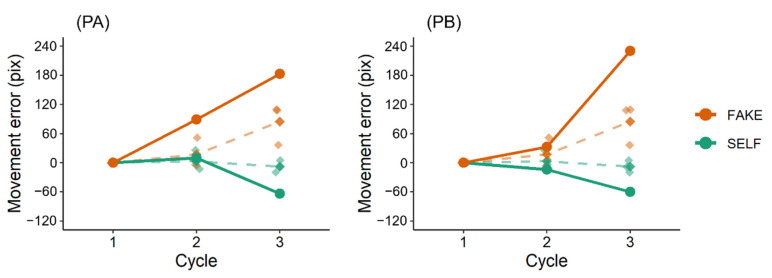
Movement errors. Rhombus plots and dashed lines show movement errors of healthy participants (mean and individual values). Movement errors in all participants were smaller in the SELF condition (−63.31–4.91 pix) than in the FAKE condition (36.76–230.19 pix).

A mean movement error for each cycle was calculated. Because the movement error in Cycle 1 served as the baseline for those in Cycles 2 and 3, that in Cycle 1 was subtracted from that in each cycle, so that in each condition, it was zero at Cycle 1. The results (Figure 3) showed that movement errors in all participants were smaller in the SELF condition (−63.31–4.91 pix) than in the FAKE condition (36.76–230.19 pix).

### 3.2. Self-Other Judgment

**Figure 4:**
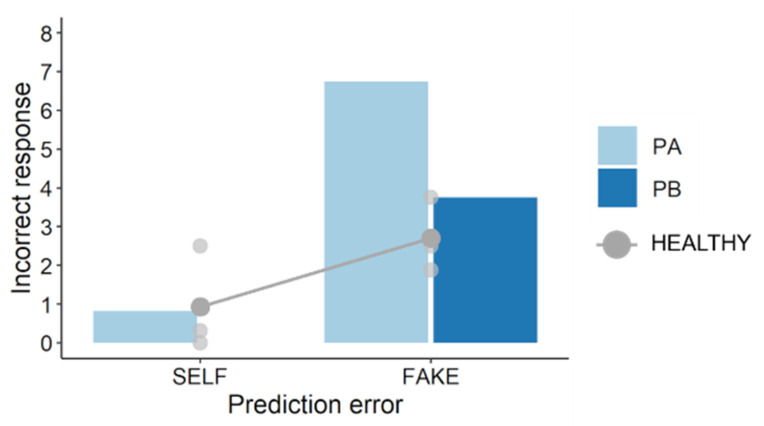
Incorrect responses in self-other judgments. In the FAKE condition, the incorrect response score in PA (6.75) was higher than that in PB (3.75) or healthy participants (3.75; 2.50; 1.88). PB (and one healthy participant) made no incorrect responses in the SELF condition. Grey line shows the mean score in healthy participants.

To quantify participants’ incorrect responses (i.e., subjective misattributions), we calculated the differences between the correct scores (nine for the SELF condition and one for the FAKE condition) and the actual mean score in each condition. Specifically, we subtracted the mean score from nine for the SELF condition and subtracted one from the mean score for the FAKE condition; thus, incorrect response scores were positive. The results (Figure 4) showed that in the FAKE condition, the incorrect response score in PA (6.75) was higher than that in PB (3.75) or healthy participants (3.75; 2.50; 1.88).

### 3.3. Prediction Error and Self-Other Judgment

**Figure 5:**
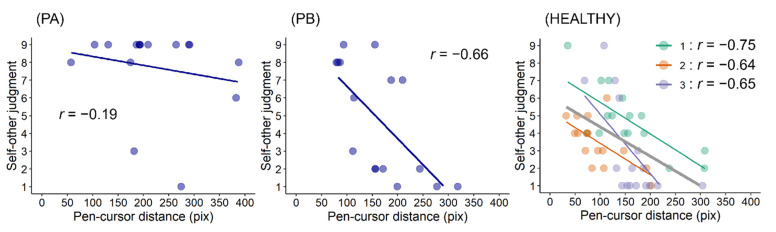
Prediction errors and self-other judgments in the FAKE condition. Self-other judgment refers to a nine-point scale ranging from nine (completely self-movement) to one (completely other’s movement). Moderate or high correlations were observed in PB (*r* = −0.66) and three healthy participants (*r* = −0.75; *r* = −0.64; *r* = −0.65), whereas in PA, a low correlation was observed, *r* = −0.19.

Correlation analyses revealed that moderate or high correlations [23] were observed in PB (*r* = −0.66, *p* = 0.005) and three healthy participants (*r* = −0.75, *p* < 0.001; *r* = −0.64, *p* = 0.008; *r* = −0.65, *p* = 0.007), whereas in PA, a low correlation [23] was observed, *r* = −0.19, *p* = 0.48 (Figure 5). A summary of the results is shown in Table 2.

## 4. Discussion

This study preliminarily investigated whether post-stroke sensorimotor deficits can impair the link between prediction error and self-other attribution. In this task, movement errors in the SELF condition should be smaller than those in the FAKE condition if participants perform feedback control of the cursor movement [10]. The results reflected this, suggesting that participants correctly completed this movement task. Regarding self-other judgments, the results showed that PA made more incorrect self-attributions of prerecorded movements (i.e., FAKE condition) than PB and healthy participants. PA had worse upper limb sensorimotor deficits and lesser paretic upper limb activity than PB. Therefore, these results are consistent with those of the previous studies [13,14], suggesting that post-stroke sensorimotor deficits can alter self-other attribution. Importantly, these results also showed that self-other judgment scores in PA were weakly correlated with pen–cursor distances (i.e., prediction errors), whereas those in PB and healthy participants were moderately or strongly correlated with them. This finding indicates that post-stroke sensorimotor deficits can impair the relationship between prediction error and self-other attribution, and therefore supports the hypothesis, proposed in previous studies [13,14], that such deficits can result in altered strategies for self-other attribution.

Based on the cue integration theory [2,15], in our previous studies [13,14], we proposed that in patients with post-stroke sensorimotor deficits, sensorimotor cues can be less weighted because of their deficits; therefore, other cognitive cues, such as thought or knowledge, may be more weighted (i.e., altered strategies for self-other attribution). This study did not focus on the effects of cognitive cues but provided evidence regarding the weight of sensorimotor cues; the weak correlation between prediction errors and self-other judgments in PA suggests that the sensorimotor cues (i.e., prediction error) were less weighted. This can be interpreted to mean that his prediction error tolerance for the registration of agency was wider than that in PB or healthy participants. If pen–cursor distances had been longer than 400 pix (see Figure 5), PA might have attributed a fake movement to the other (i.e., reduction of misattributions). Although the threshold of prediction error is unclear, post-stroke sensorimotor deficits can lower the weight of sensorimotor cues, resulting in a wide prediction error tolerance.

This study had several limitations. First, PA and PB had a lesion on different sides of the hemispheres. A lesion side on particular cortices or their network, such as frontal or parietal regions, may be involved in altered agency [24,25]. However, lesion sites in PA and PB were subcortical, and both had a lesion in similar regions (e.g., thalamus or corona radiata), although their sides were contrasted. Further studies are needed to investigate the association between subcortical lesions and altered agency. Second, since patients performed this task using their non-paralyzed upper limb, PA and healthy participants used their right upper limb, whereas PB used his left one. Third, healthy participants’ ages did not match those of PA and PB. These differences could have affected the results. However, the previous study confirmed that there were no significant differences in the results between the right and left upper limb uses [13]. Regarding the age mismatch, the age of PA matched that of PB, but only results of PB were similar to those of healthy participants; the results in PA are unlikely to be explained by the age difference. Considering these facts, we concluded that the altered self-other attribution in PA resulted from worse sensorimotor deficits and lesser activity in his paretic upper limb compared with PB. Finally, the findings of this study were based on a sample of only two patients and three healthy adults. This study was conducted as a preliminary experiment; therefore, the findings should be further investigated in future studies.

## 5. Conclusions

This study suggests that post-stroke sensorimotor deficits can impair the linkage between prediction error and self-other attribution, supporting the hypothesis that patients with such deficits can have altered strategies for self-other attribution [13,14]. Understanding these strategies may be important for clinical practices. For example, even if one assisted a patient’s movement so that the movement outcome matches the motor command (i.e., assistance in lessening the prediction error), the patient might make self-other attribution based on cues other than prediction error. This may result in altered agency, such as a loss of agency or misattribution. Additionally, given that the sense of agency plays an important role in motor control [3,10], interventions that consider the patient’s strategies may be beneficial in rehabilitation. The present study underscores the need for further studies that investigate what cues such patients utilize in their self-other attribution other than sensorimotor cues (i.e., prediction error), which can contribute to revealing the strategies for self-other attribution in patients with post-stroke sensorimotor deficits.

## Figures and Tables

**Table 1:** Patients’ clinical characteristics.

	Patient A	Patient B
Age	70s	60s
Sex	Male	Male
Stroke type	Hemorrhage	Hemorrhage
Paretic side	Left	Right
FMA-UE	51	61
ARAT	28	53
Proprioception	4	4
Tactile	2	2
AOU	2.5	3.4
QOM	2.0	4.0
MMSE	30	30
TMT-A	62	84
K-AST	12	12
CBS	0	0

FMA-UE refers to the total score of the FMA-UE motor-related subscales. Proprioception refers to the total score of the FMA-UE proprioception subscales. Tactile refers to the total score of the FMA-UE tactile subscales. FMA-UE: Fugl-Meyer Assessment of upper extremity; ARAT: Action Research Arm Test; AOU: amount of use; QOM: quality of movement; MMSE: Mini Mental State Examination; TMT-A: Trail-Making Test A; K-AST: Korean version of apraxia screen of TULIA; CBS: Catherine Bergego Scale.

**Table 2:** A summary of self-other attribution results.

	Incorrect Response		Correlation between Prediction Errors and Self-Other Judgments
	SELF	FAKE	
Patient A	0.81	6.75	−0.19
Patient B	0.00	3.75	−0.66
Healthy participant 1	0.00	3.75	−0.75
Healthy participant 2	2.50	2.5	−0.64
Healthy participant 3	0.31	1.88	−0.65

## Data Availability

The original contributions presented in the study are included in the article. Further inquiries can be directed to the corresponding author.
